# Newly Isolated *Paenibacillus tyrfis* sp. nov., from Malaysian Tropical Peat Swamp Soil with Broad Spectrum Antimicrobial Activity

**DOI:** 10.3389/fmicb.2016.00219

**Published:** 2016-03-01

**Authors:** Yoong-Kit Aw, Kuan-Shion Ong, Learn-Han Lee, Yuen-Lin Cheow, Catherine M. Yule, Sui-Mae Lee

**Affiliations:** ^1^Tropical Biology Multidisciplinary Platform, School of Science, Monash University MalaysiaBandar Sunway, Malaysia; ^2^School of Science, Monash University MalaysiaBandar Sunway, Malaysia; ^3^Jeffrey Cheah School of Medicine and Health Sciences, Monash University MalaysiaBandar Sunway, Malaysia

**Keywords:** *Paenibacillus*, antimicrobial, peat swamp, Malaysia, lipopeptide

## Abstract

Emergence of antimicrobial resistance coupled with the slowdown in discovery of new antimicrobial compounds points to serious consequences for human health. Therefore, scientists are looking for new antimicrobial compounds from unique and understudied ecosystems such as tropical peat swamp forests. Over the course of isolating antimicrobial producing bacteria from North Selangor tropical peat swamp forest, Malaysia, a Gram variable, rod shaped, endospore forming, facultative anaerobic novel strain MSt1^T^ that exerts potent and broad spectrum antimicrobial activity was isolated. Phylogenetic analysis using 16S rRNA gene sequences showed that strain MSt1^T^ belonged to the genus *Paenibacillus* with the highest similarity to *Paenibacillus elgii* SD17^T^ (99.5%). Whole genome comparison between strain MSt1^T^ with its closely related species using average nucleotide identity (ANI) revealed that similarity between strain MSt1^T^ with *P. elgii* B69 (93.45%) and *Paenibacillus ehimensis* A2 (90.42%) was below the recommended threshold of 95%. Further analysis using *in silico* pairwise DDH also showed that similarity between strain MSt1^T^ with *P. elgii* B69 (55.4%) and *P. ehimensis* A2 (43.7%) was below the recommended threshold of 70%. Strain MSt1^T^ contained *meso*-diaminopilemic acid in the cell wall and MK-7 as the major menaquinone. The major fatty acids of strain MSt1^T^ were anteiso-C_15:0_ (48.2%) and C_16:0_ (29.0%) whereas the polar lipid profile consisted of phosphatidylglycerol, phosphatidylethanolamine, diphosphatidylglycerol, one unknown lipid, two unknown glycolipids, and one unknown phospholipid. Total DNA G+C content of strain MSt1^T^ was 51.5 mol%. The extract from strain MSt1^T^ exerted strong antimicrobial activity against *Escherichia coli* ATCC 25922 (MIC = 1.5 μg/mL), MRSA ATCC 700699 (MIC = 25 μg/mL) and *Candida albicans* IMR (MIC = 12.5 μg/mL). Partially purified active fraction exerted a strong effect against *E. coli* ATCC 25922 resulting in cell rupture when viewed with SEM. Based on distinctive taxonomic differences between strain MSt1^T^ when compared to its closely related type species, we propose that strain MSt1^T^ represents a novel species within the genus of *Paenibacillus*, for which the name *Paenibacillus tyrfis* sp. nov. (= DSM 100708^T^ = MCCC 1K01247^T^) is proposed.

## Introduction

Antimicrobial resistant bacteria (ARB) are a major concern because infections by ARB are directly responsible for not only increased healthcare costs but also high mortality rates (Boucher et al., 2009; Davies and Davies, 2010). Infections by ARB cost approximately $55 billion annually in the United States (Smith and Coast, 2013). Currently, more than 70% of nosocomial pathogens are resistant to at least one of the antimicrobials commonly used to treat infections caused by them (Mishra et al., 2012). The rapid emergence of ARB compounded with the decline in new antimicrobial discoveries further complicates the problem. For example, two new antimicrobials that have been clinically approved in the past decade are linezolid and daptomycin. However, bacteria resistant to these antimicrobials were already reported shortly after their introduction (Meka and Gold, 2004; Hayden et al., 2005). Therefore, there is a dire need to discover new antimicrobials to combat ARB related infections.

One strategy to look for novel bioactive compounds is bioprospecting in extreme, understudied ecosystems. The discovery of abyssomycin produced by *Verrucosispora* spp. isolated from South China Sea sediment is an example of successful bioprospecting (Bister et al., 2004). Bacteria produce a wide array of compounds to obtain a competitive advantage by suppressing their competitors and to colonize new habitats. Bacteria capable of thriving in such extreme conditions are very likely to evolve special adaptations and produce unique molecules or compounds that may have beneficial uses (Kohama et al., 1993; Pettit, 2011).

Indo-Malaysian tropical peat swamp forests are an example of a unique ecosystem, characterized by the formation of deep layers of peat in an acidic, waterlogged, nutrient poor environment. Despite such a challenging habitat, there is an enormous microbial diversity in tropical peat swamp forests. Kanokratana et al. (2011) performed a metagenomic study on Thai tropical peat soil and showed that 80% of the bacterial community could be potentially novel species or strains. The North Selangor tropical peat swamp forest in Malaysia was therefore chosen to be the site of study and during the course of isolating antimicrobial producing bacteria, a novel bacteria strain MSt1^T^ was isolated (Aw et al., 2014). Strain MSt1^T^ exerts broad spectrum antimicrobial activity against many clinically relevant pathogens such as methicillin resistant *Staphylococcus aureus* (MRSA), vancomycin resistant *Enterococcus* (VRE), *Escherichia coli, Pseudomonas aeruginosa, Salmonella* Typhimurium, *Candida albicans* and *Cryptococcus neoformans* to name a few. Further analysis revealed that strain MSt1^T^ belonged to the genus *Paenibacillus*.

The genus *Paenibacillus* was proposed by Ash et al. (1993) to distinguish members of the “16S rRNA group 3″ bacilli from other members in the genus *Bacillus*. Currently, there are 165 species in the genus *Paenibacillus* and the type species is *Paenibacillus polymyxa*. Members of *Paenibacillus* are known to be Gram variable, rod shaped, aerobic or facultatively anaerobic, endospore forming and motile via peritrichous flagella. DNA G+C content of *Paenibacillus* species is between 39 and 54 mol%, the major cellular fatty acid is anteiso-C_15:0_, the cell wall peptidoglycan diamino acid is *meso*-diaminopimelic acid and the major menaquinone is menaquinone-7 (MK-7; Shida et al., 1997). Members belonging to the genus *Paenibacillus* are well known to produce a wide range of antimicrobial compounds e.g., *P. ehimensis* that produces a cyclic lipopeptide (Aktuganov et al., 2008; Huang et al., 2013), *P. koreensis* that produces an iturin-like antifungal (Chung et al., 2000), *P. elgii* that produces pelgipeptins (Kim et al., 2004; Wu et al., 2010; Ding et al., 2011) and *P. polymyxa* that produces polymyxin B (Paulus and Gray, 1964; Shaheen et al., 2011).

In this study, *Paenibacillus* sp. strain MSt1^T^ was subjected to a polyphasic taxonomic approach as described by Logan et al. (2009) with respect to the minimal standards for description of aerobic, endospore-forming bacteria and results indicated that strain MSt1^T^ represented a novel species within the genus *Paenibacillus*, for which the name *Paenibacillus tyrfis* sp. nov. is proposed. The antimicrobial activity of the extract produced by strain MSt1^T^ was characterized and chemical analysis was performed to determine the constituents present in the extract.

## Materials and methods

### Isolation and maintenance of the isolate

Strain MSt1^T^ was isolated as described in Aw et al. (2014). Briefly, strain MSt1^T^ was isolated from North Selangor tropical peat swamp soil (3° 39′ 30.8″ N; 101° 19′ 18.4″ E) from surface peat soil. Strain MSt1^T^ was selected for further study due to its wide spectrum antimicrobial activity against these indicator pathogens. Pure cultures of strain MSt1^T^ were routinely maintained on TSA at 30°C and long term storage was performed via cryopreservation with 25% (v/v) glycerol at −80°C.

### 16S rRNA gene analysis and phylogenetic analysis

Genomic DNA was extracted from a 3 day old culture of strain MSt1^T^ on TSA using GF-1 DNA extraction kit (Vivantis, Malaysia) according to manufacturer's instructions. The almost complete 16S rRNA gene sequence of strain MSt1^T^ (1492 bp; DDBJ/EMBL/GenBank accession number KT216503) was amplified according to Lee et al. (2014) using the primer pairs of 27f and 1492r. Briefly, 16S rRNA gene sequence was amplified using universal primer pair 27f (5′ AGAGTTTGATCMTGGCTCAG 3′) and 1492r (5′ TACGGYTACCTTGTTACGACTT 3′). PCR reaction mixture contained 5 × MyTaq Red Buffer, 1.25 U MyTaq DNA polymerase (Bioline, United Kingdom), 0.5 μM of 27f forward primer, 0.5 μM of 1492r reverse primer and 3 μL of DNA template with the final volume of 50 μL. The thermal cycling profile of initial denaturation at 95°C for 5 min followed by 30 cycles of denaturation at 95°C for 1 min, annealing at 55°C for 1 min and elongation at 72°C for 1 min were used.

The resulting 16S rRNA gene sequence was compared to the sequences in the EzTaxon database (http://eztaxon-e.ezbiocloud.net/; Kim et al., 2012). The 16S rRNA gene sequence of strain MSt1^T^ was aligned with sequences of closely related type species that had been retrieved from the GenBank/EMBL/DDBJ databases using CLUSTAL-X software (Thompson et al., 1997). The alignment was manually verified and adjusted prior to the construction of the phylogenetic tree using the neighbor-joining algorithm (Saitou and Nei, 1987) with the MEGA version 6 software (Tamura et al., 2013). The EzTaxon-e server (http://eztaxon-e.ezbiocloud.net/; Kim et al., 2012) was used for calculations of sequence similarity level. The stability of the resultant tree topologies were evaluated by using the bootstrap resampling method (1000 resampling) of Felsenstein (1985). The evolutionary distances were computed using Kimura's two-parameter model (Kimura, 1980). The 16S rRNA gene sequence of strain MSt1^T^ was deposited at DDBJ/EMBL/GenBank under the accession no. KT216503.

### Whole genome sequencing, average nucleotide identity (ANI) and *in silico* pairwise DNA-DNA hybridization (DDH)

The draft genome sequence of strain MSt1^T^ was obtained as described in Aw et al. (2014). The draft genome of strain MSt1^T^ was deposited at DDBJ/EMBL/GenBank under the accession no. JNVM00000000.

As the 16S rRNA gene sequence of strain MSt1^T^ showed more than 97% similarity when compared with its closely related type species, analyses that compare between genomes of closely related species such as ANI and DDH using the draft genome sequence of strain MSt1^T^ were performed. ANI determination was performed at EzGenome (http://www.ezbiocloud.net/ezgenome/ani) as described by Goris et al. (2007) by comparing the draft genome of strain MSt1^T^ to its closely related isolates as determined from the phylogenetic analysis of the 16S rRNA sequences. Estimation of *in silico* pairwise DDH was performed with genome to genome distance calculator (GGDC 2.0; ; Meier-Kolthoff et al., 2013).

### Phenotypic characteristics

Phenotypic analysis was performed using growths on TSA at 30°C for 3 days unless otherwise mentioned. Colony morphology was determined using growths on TSA at 30°C for 3 days. Cellular morphology (vegetative cells, sporulating cells, and flagellation) was observed using a light microscope (Olympus EX43) and scanning electron microscopy (Hitachi SU8010) following growth in tryptone soy broth (TSB) at 30°C for 5 days (Figure S1). Sporulation was promoted by growing the strain on TSB supplemented with 10 μg/mL MnSO_4_ at 30°C for 5 days and stained with Schaeffer-Fulton stain (Schaeffer and Fulton, 1933). Gram staining was performed using standard Gram staining procedure and Gram reaction was confirmed using the KOH lysis test (Cerny, 1978; Moaledj, 1986). Flagellation was determined using Ryu's flagella stain (Kodaka et al., 1982). Motility was determined using semi-solid agar as described by Tittsler and Sandholzer (1936).

The following phenotypic tests were performed as described by Wu et al. (2011) and Glaeser et al. (2013). Optimal temperature study was performed using growth in TSB at 4, 15, 25, 30, 37, 42, and 50°C for 7 days. Optimal pH for growth was studied with pH between 3 and 10 (1 pH unit interval) and salt tolerance was determined with 0–5% (w/v) NaCl (1% intervals) using TSB at 30°C for 7 days. Catalase activity was tested by bubble production with 3% (v/v) hydrogen peroxide solution (Merck). Oxidase activity was tested with color change using 1% tetramethyl-*p*-phenylenediamine (BD). Acid production from a sole carbon source were determined using API 50CH (bioMérieux) according to the manufacturer's instructions. API ZYM and API 20E (bioMérieux) strips were used to determine enzyme activity of strain MSt1^T^.

### Chemotaxonomic characteristics

For the chemotaxonomic approach, analyses of polar lipid, respiratory quinone, cellular fatty acids, and cell wall peptidoglycan were performed by Identification Service of DSMZ (Braunschweig, Germany). Biomass of strain MSt1^T^ was grown in TSB at 30°C for 5 days. Polar lipids and major respiratory quinones were extracted and analyzed using thin layer chromatography (TLC) as described by Kates (1986). Fatty acid analysis was performed according to Miller (1982) and Kuykendall et al. (1988). Biomass of strain MSt1^T^ and its reference species were grown in TSB at 30°C for 5 days. Determination of cell wall peptidoglycan was performed according to Rhuland et al. (1955) with the freeze-dried biomass of strain MSt1^T^, grown in TSB at 30°C for 5 days.

### Preparation of partially purified extract from strain MSt1^T^

Partial purification of extract produced by strain MSt1^T^ was performed according to Ding et al. (2011) with modification. Briefly, strain MSt1^T^ was grown on TSA for 3–4 days at 30°C. Whole agar including the bacteria was placed into a glass jar and extraction was performed three times using 100% acetonitrile (Merck, Germany). The extract was concentrated under reduced pressure using a rotary evaporator and it was then freeze-dried. The dried crude extract was then packed into a C-18 (Merck) column and eluted with 50:50, 70:30, and 100:0% methanol:water. The active fraction (100% methanol) was collected and concentrated under reduced pressure using a rotary evaporator followed by freeze-drying. The active fraction was further fractionated using a preparative HPLC system [Agilent Infinity 1260 Quaternary LC system (Agilent, USA)] with a reverse phase column [Purospher STAR RP-18 endcapped (Merck), 4.6 μm, 250 × 20 mm]. Mobile phase A consisted of 100% Milli-Q water and mobile phase B consisted of 100% methanol. A linear gradient of 60–95% B in 15 min at a flow rate of 18 mL/min were used as elution and monitored at 254 nm. All isolated peaks were collected and the active fraction was evaporated and stored at -20°C for further analysis.

### Minimum inhibitory concentration (MIC) determination of active fraction from strain MSt1^T^

Antimicrobial activity of the active fraction from strain MSt1^T^ was tested against *E. coli* ATCC 25922, *P. aeruginosa* ATCC 10145, methicillin sensitive *S. aureus* (MSSA) ATCC 29213, MRSA 700699, VRE ATCC 700802, and *C. albicans* from IMR (Institute for Medical Research, Malaysia) via the broth microdilution method as described by CLSI (2012). Briefly, pathogens were grown overnight in brain heart infusion broth (BHI) and adjusted to 0.5 McFarland standard (OD_625_ at 0.08–0.13). This adjusted culture was diluted 100 times before being used as the inoculum for broth microdilution. The active fraction was reconstituted in phosphate buffered saline (PBS; Oxoid, UK) at 50 mg/mL and serially diluted in the 96-well microtitre plate (SPL Life Science, Korea) using sterile BHI broth. Diluted inoculum (100 μL) was added to the microtiter plate. Positive control used against bacteria was 1.25 mg/mL chloramphenicol (Nacalai-Tesque, Japan) whereas positive control used against *C. albicans* was 5 mg/mL cycloheximide (Nacalai-Tesque). The microtitre plate was incubated at 37°C for 18–24 h. All MIC determination were performed in triplicates. MIC was determined by the lowest concentration of active fraction where no visible growth was observed in the well according to CLSI (2012).

### Effect of active fraction on cellular morphology via scanning electron microscopy (SEM)

The mode of action of the active fraction produced by strain MSt1^T^ was studied by looking at the effect of the antimicrobial compound on the surface structure of the *E. coli* ATCC 25922. The cellular structure of *E. coli* after treatment with the active fraction was examined using field emission scanning electron microscopy (FE-SEM). Briefly, *E. coli* was grown in BHI at 37°C for 18 h. The culture was adjusted to McFarland 0.5 (OD_625_ at 0.08–0.13) and a final concentration of 0.1 mg/mL of active fraction was added and incubated at 37°C for 4 h. The culture was centrifuged at 5000 × *g* for 3 min and the supernatant was removed. The pellet was washed with PBS (Oxoid) and subjected to centrifugation at 5000 × *g* for 3 min. This washing process was performed three times. The washed pellet was reconstituted with minimal volume of PBS, placed onto a glass slide and allowed to air dry for 30 min.

The slide was fixed using 2.5% (v/v) gluteraldehyde (Sigma, USA) in PBS overnight and washed three times using PBS. The slide was then subjected to serial dehydration from 20% ethanol to 100% ethanol for 10 min on each step and kept in a desiccator overnight. The slide was spur-coated with platinum using Q150R Rotary-Pumped Sputter Coater before being observed using Hitachi SU8010 FE-SEM.

## Results and discussion

### 16S rRNA gene sequence and phylogenetic analyses

**Figure 1:**
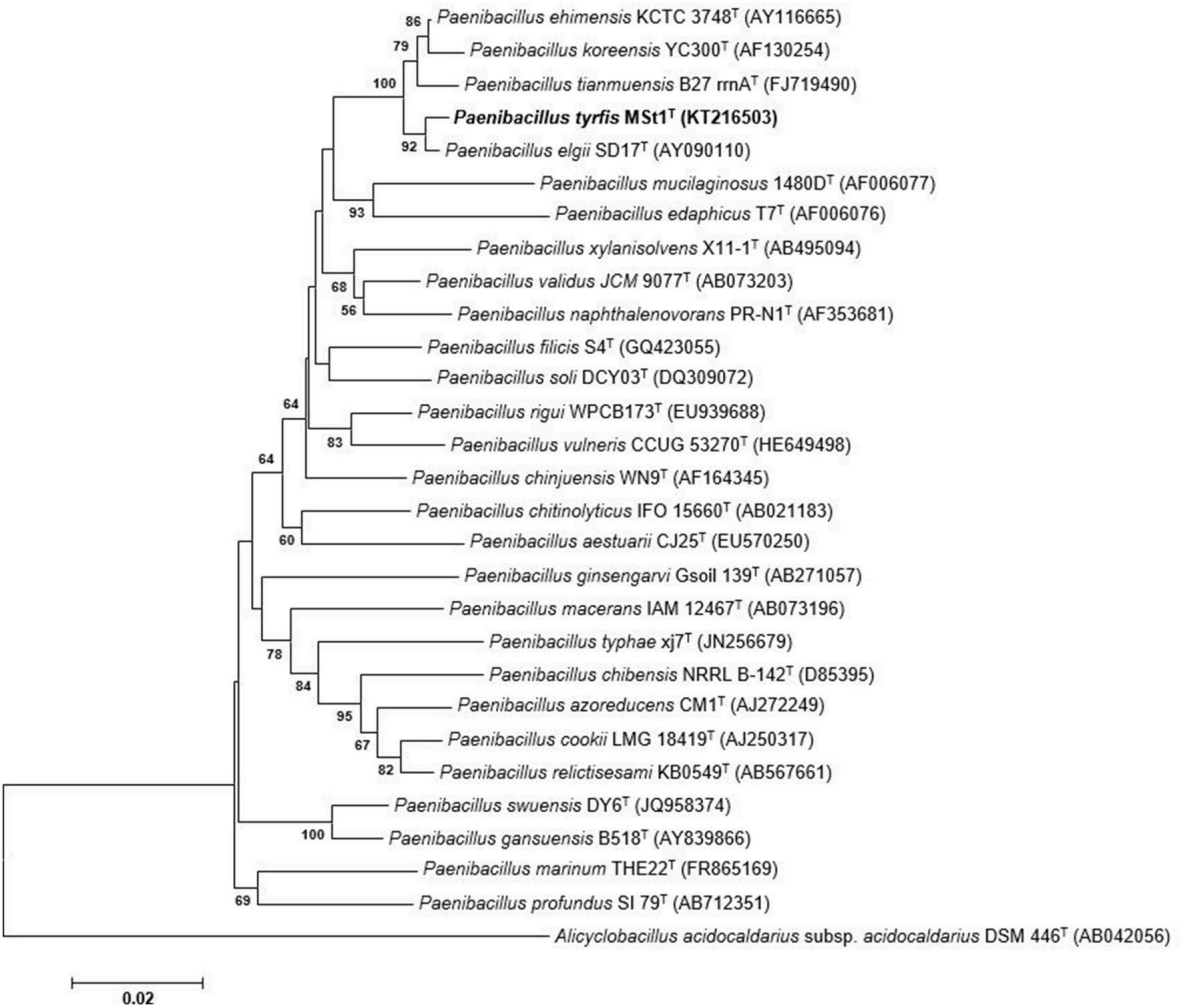
**Neighbor-joining tree based on 16S rRNA sequences showing relationships between strain MSt1^T^ and representatives of other related taxa**. Bootstrap values (>50%) based on 1000 resampled datasets are shown at branch nodes. *Alicyclobacillus acidocaldarius* subsp. *acidocaldarius* DSM 446^T^ was used as the outgroup. Bar, 2 substitutions per 100 nucleotide positions.

The 16S rRNA gene sequence of strain MSt1^T^ was found to be similar to *P. elgii* SD17^T^ (99.5%), *P. ehimensis* KCTC 3748^T^ (98.8%), and *P. tianmuensis* B27^T^ (98.2%), whereas sequences similarities of less than 97.9% were obtained to other *Paenibacillus* species. Phylogenetic trees were constructed to determine the phylogenetic position of this strain (Figure 1). The phylogenetic analysis revealed that strain MSt1^T^ was closely related to the type strain *P. elgii* SD17^T^ as they formed a distinct clade supported by a high bootstrap value (94%), indicating a high confidence level of this association (Figure 1). The closest related species, *P. elgii* SD17^T^, *P. ehimensis* KCTC 3748^T^, and *P. tianmuensis* B27^T^ were used as reference strains for further polyphasic analysis.

### Whole genome sequence, ANI, and *in silico* pairwise DDH analyses

The draft genome sequence of strain MSt1^T^ was reported in Aw et al. (2014). As the 16S rRNA gene sequence of strain MSt1^T^ showed more than 97% similarity when compared with its closely related type species, alternative methods such as DDH should be performed as recommended by Tindall et al. (2010). With the advance in genomics and whole genome sequencing, *in lieu* of DDH, direct comparison between genome sequences between strains can be performed with methods such as ANI and *in silico* pairwise DDH. Using the draft genome of strain MSt1^T^ and compared with *P. elgii* B69 and *P. ehimensis* A2, the ANI value were 93.45 and 90.42%, respectively. The threshold of 70% genomic relatedness with DDH is generally recommended for species delineation (Wayne et al., 1987) and has been found to correlate to 95–96% average nucleotide identity (ANI; Goris et al., 2007; Richter and Rosselló-Móra, 2009; Kim et al., 2014). As the ANI value obtained was below the 95–96% threshold, this supports the proposal that strain MSt1^T^ belongs to a novel species.

Further analyses using the draft genome sequence were performed using *in silico* pairwise DDH determination. Using the draft genome of strain MSt1^T^ and compared with *P. elgii* B69 and *P. ehimensis* A2, the *in silico* pairwise DDH value was estimated to be 55.40 ± 2.73% and 43.70 ± 2.54%, which was below the recommended threshold of 70% as recommended by Wayne et al. (1987). Therefore, the results of ANI and *in silico* DDH both agreed that strain MSt1^T^ represents a new species at the genome level.

### Phenotypic characteristics

Strain MSt1^T^ was found to be Gram variable rods, facultatively anaerobic, endospore-forming, and motile via peritrichous flagella. Colonies grown on TSA are milky white, round, sticky colonies. Colonies are approximately 5–8 mm in diameter after incubation for 3 days at 30°C on TSA. Cells are approximately 3–5 μm in length and 0.6–1.0 μm in width. Ellipsoidal endospores were found in a terminal position. The growth temperature ranges from 20 to 42°C with an optimal temperature of 30°C. Growth occurs between pH 3 and 10 (optimum at pH 6–7) and between 0 and 2% (w/v) NaCl (optimum growth at 1%). Strain MSt1^T^ is both catalase, oxidase and haemolysis positive.

The physiological and biochemical characteristics of strain MSt1^T^ were compared with its most closely related type strains (Table 1) and showed distinctive differences from these type strains. Strain MSt1^T^ was found to show distinctive differences as compared to its closely related type strains in enzymatic reaction of α-glucosidase and β-glucosidase together with the inability to produce indole and ferment L-arabinose.

**Table 1:** **Distinctive phenotypic characteristics of strain MSt1^T^ when compared to its closely related type species of *Paenibacillus***.

**Characteristics**	**MSt1^T^**	***P. elgii* SD17^T^**	***P. ehimensis* KCTC 3748^T^**	***P. tianmuensis* B27^T^**
Gram stain	v	V	v	v
Motility	+	+	+	+
Catalase	+	+	+	+
Oxidase	+	–	+	+
Anaerobic growth	+	+	+	+
α-glucosidase	–	+	+	+
β-glucosidase	–	+	+	+
Protease (trypsin)	–	V	+	+
Urease	–	+	–	–
Growth at 50°C	–	–	+	+
Indole production	–	+	–	–
**FERMENTATION OF:**				
L-arabinose	–	–	+	–
D-xylose	+	V	+	+
DNA G+C content (%)	51.5	51.7	54.9	47

Data were obtained from this study except for DNA G+C content (%) which were obtained from Kim et al. (2004), Lee et al. (2004) and Wu et al. (2011). (+): positive, (–): negative (v): variable.

### Chemotaxonomic analyses

The polar lipid profile of strain MSt1^T^ consisted of phosphatidylglycerol (PG), phosphatidylethanolamine (PE), diphosphatidylglycerol (DPG), one unknown lipid, two unknown glycolipids and one unknown phospholipid (Figure S2). Members of the genus *Paenibacillus* are known to have DPG as the major polar lipid with some species containing PE and PG. The major respiratory quinones of strain MSt1^T^ was found to be MK-7 which is characteristic of members of *Paenibacillus* (Shida et al., 1997).

The major fatty acid found in strain MSt1^T^ was anteiso-C_15:0_ (48.2%) and C_16:0_ (29.0%; Table 2). The fatty acid profile of strain MSt1^T^ was found to be similar to the reference strains where all three reference species also contained anteiso-C_15:0_ (39.9–50.0%) and C_16:0_ (22.0–37.0%) as their major fatty acid. The main difference between strain MSt1^T^ and *P. elgii* SD17^T^ was that strain MSt1^T^ was nearly double that of anteiso-C_15:0_as compared to C_16:0_ whereas *P. elgii* SD17^T^ contained almost equal amounts of both anteiso-C_15:0_ and C_16:0_. It was also found that the cell wall peptidoglycan of strain MSt1^T^ was *meso*-diaminopimelic acid which was consistent with other species of the genus *Paenibacillus* (Shida et al., 1997).

**Table 2:** **Cellular fatty acid profile of strain MSt1^T^ when compared to its closely related type species of *Paenibacillus***.

**Fatty acid**	**MSt1^T^**	***P. elgii* SD17^T^**	***P. ehimensis* KCTC 3748^T^**	***P. tianmuensis* B27^T^**
**SATURATED**				
C_14:0_	7.4	5.8	5.2	4.8
C_16:0_	29.0	37.1	28.4	22.0
**BRANCHED**				
iso-C_15:0_	4.4	5.0	6.3	10.4
anteiso-C_15:0_	48.2	40.0	45.5	49.9
iso-C_16:0_	2.6	2.1	4.1	2.5
iso-C_17:0_	1.5	3.0	2.80	3.5
anteiso-C_17:0_	5.1	5.4	5.4	5.1

Data were obtained from this study. Values < 1% was omitted.

In view of morphological, physiological, chemotaxonomic, phylogenetic and genomic results obtained for strain MSt1^T^, it is evident that strain MSt1^T^ belongs to the genus *Paenibacillus*. Distinct differences in physiological, chemotaxonomic and genomic data between strain MSt1^T^ and its closely related type species make it evident that strain MSt1^T^ represent a novel species for which the name *P. tyrfis* sp. nov. is proposed.

### Antimicrobial activity of active fraction from strain MSt1^T^

The active fraction from strain MSt1^T^ showed strong antimicrobial effects against all six pathogens tested (Table 3). The active fraction was found to be more effective against Gram negative bacteria such as *E. coli* (MIC = 1.5 μg/mL) and yeasts such as *C. albicans* (MIC = 12.5 μg/mL) as compared to Gram positive bacteria such as *E. faecalis* (MIC = 50 μg/mL).

**Table 3:** **MIC of active fraction produced by strain MSt1^T^ against six pathogens**.

**Pathogen tested**	**MIC (μg/mL)**
*Escherichia coli* ATCC 25922	1.5
*Pseudomonas aeruginosa* ATCC 10145	12.5
MSSA ATCC 29213	25
MRSA ATCC 700699	25
VRE ATCC 700802	50
*Candida albicans* IMR	12.5

**Figure 2:**
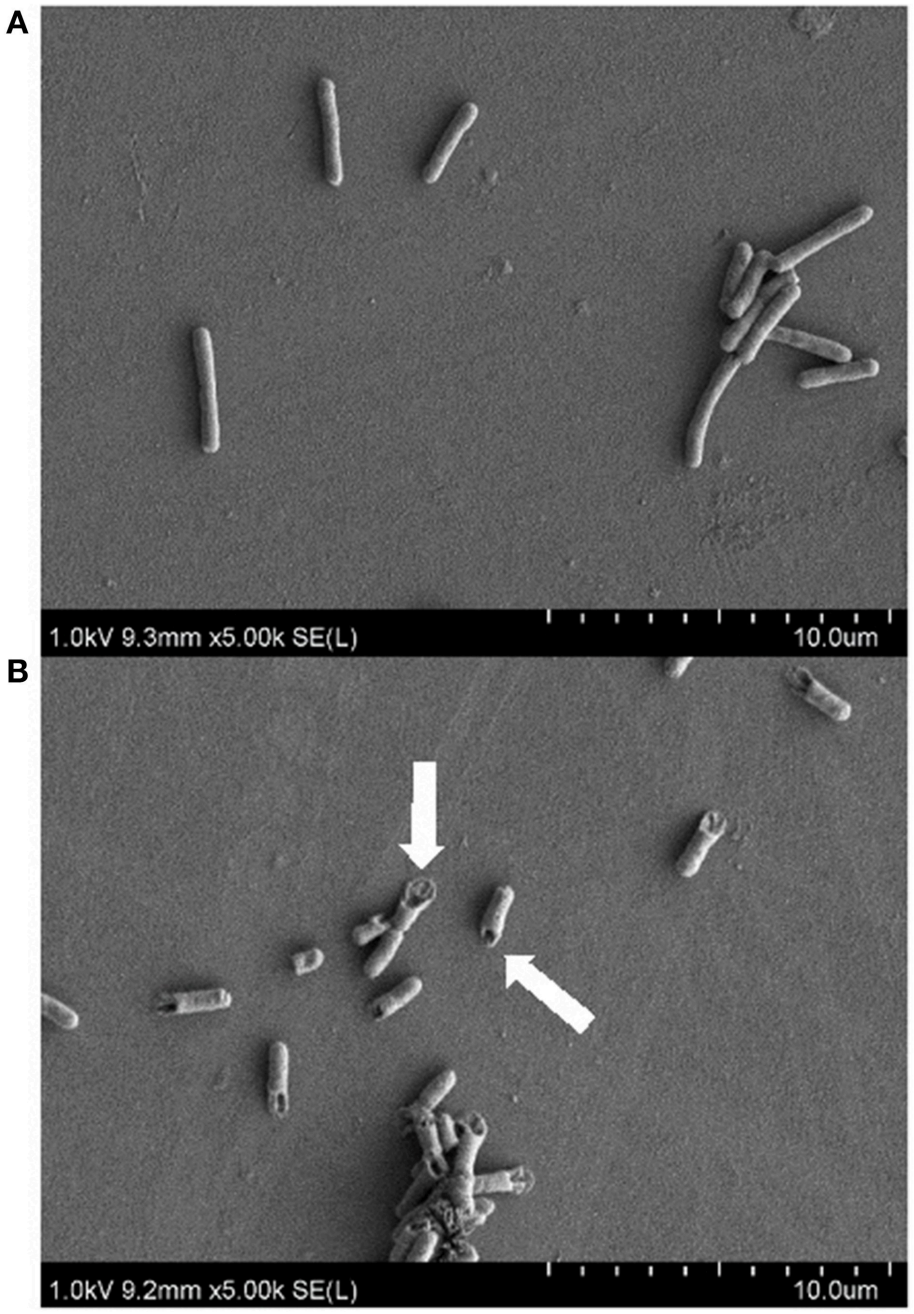
**Scanning electron microscopy images on the antimicrobial effect of active fraction produced by strain MSt1^T^ against *E. coli* ATCC 25922 at 5000x magnification**. **(A)** Negative control (treatment with PBS); **(B)** Treatment with 0.1 mg/mL of active fraction produced by strain MSt1^T^. Arrows points at the cell rupture of *E. coli* after treatment.

As the active fraction produced by strain MSt1^T^ was found to be most effective against *E. coli*, SEM imaging was used to study the antimicrobial effect on the surface morphology of *E. coli. E. coli* treated with 0.1 mg/mL of the active fraction resulted in cell rupture as compared to the control that was being treated with PBS (Figure 2). Cell rupture can be caused by several reasons such as disruption of cell membrane or weakening of cell wall (Thimon et al., 1995; Domenech et al., 2009; Velkov et al., 2014). Several classes of antimicrobial compounds are well known to target those sites such as glycopeptide antibiotics that disrupts the D-ala-D-ala sequence of lipid II, resulting in the disruption of transpeptidation and transglycosylation of peptidoglycan synthesis (Higgins et al., 2005; Domenech et al., 2009). This causes the weakening of the cell wall that leads to cell lysis. Another class of antimicrobial known as lipopeptide acts as a detergent that disrupts the stability of cell membrane, also leading to cell lysis (Domenech et al., 2009).

Members of *Paenibacillus* are well known to produce a wide array of antimicrobials, in particular antimicrobials belonging to the class of lipopeptides eg. polymyxin B produced by *P. polymyxa* (He et al., 2007), battacin by *Paenibacillus tianmuensis* (Qian et al., 2012) and pelgipeptin by *Paenibacillus elgii* (Wu et al., 2010; Ding et al., 2011). Preliminary chemical analysis of the active fraction using mass spectrometry revealed possible presence of lipopeptide class of antimicrobial compounds (data not shown). However, further purification of the antimicrobial is required for accurate identification of the antimicrobial compounds.

This study highlighted several key points. Although tropical peat swamp forests are known to be challenging environments for the survival of microorganisms, this study showed that tropical peat swamp forests harbor novel bacteria with the isolation and characterization of strain MSt1^T^ as *P. tyrfis*. Furthermore, strain MSt1^T^ also exhibited strong antimicrobial activity against a wide spectrum of pathogens tested; particularly the induction of cell rupture of *E. coli* when treated with the active fraction produced by strain MSt1^T^. Preliminary characterization of the active fraction highly suggests that the active fraction contains a lipopeptide-like compound. Further investigation in terms of identification and the exact mode of action of the antimicrobial compound produced by strain MSt1^T^ will be carried out.

### Description of *Paenibacillus tyrfis* sp. nov.

*P. tyrfis* (ty.r'fis *G. gen. n. tyrfi* of peat, referring to the tropical peat swamp soil, the geographical origin of the type strain).

Cells are stained Gram variable rods, facultatively anaerobic, endospore-forming, and motile via peritrichous flagella. Colonies grown on TSA are milky white, round, sticky colonies. Colonies are approximately 5–8 mm in diameter after incubation for 3 days at 30°C on TSA. Cells are approximately 3–5 μm in length and 0.6–1.0 μm in width. Ellipsoidal endospores were found in a terminal position. The growth temperature ranges from 20 to 42°C with an optimal temperature of 30°C. Growth occurs between pH 3 and 10 (optimum at pH 6 and 7) and between 0 and 2% (w/v) NaCl (optimum growth at 1%). Strain MSt1^T^ is both catalase, oxidase and haemolysis positive. Based on API ZYM test strip results, strain MSt1^T^ is positive for alkaline phosphatase, esterase (C4), esterase lipase (C8), lipase (C4), leucine arylamidase, acid phosphatse, naphthol-AS-bi-phosphohydrolase, β-glucuronidase, but negative for valine arylamidase, cysteine arylamidase, trypsin, α-chymotrypsin, α-galactosidase, β-galactosidase, α-glucosidase, β-glucosidase, n-acetyl-β-glucosaminidase, α-mannosidase, and α-fucosidase. API 20E showed that strain MSt1^T^ is positive for arginine dihydrolase, deaminase, nitrate reduction, and gelatinase, but negative for lysine decarboxylase, ornithine decarboxylase, citrate utilization, H_2_S production, urea hydrolysis, indole production and Voges-Proskauer. With API 50CH, acid is produced when strain MSt1^T^ ferments glycerol, ribose, D-xylose, D-galactose, D-glucose, D-fructose, D-mannose, D-mannitol, D-sorbitol, methyl-αD-mannopyranoside, methyl-αD-glucopyranoside, amygdaline, arbutine, esculine, salicine, D-cellobiose, D-maltose, D-lactose, D-melibiose, D-saccharose, D-trehalose, D-raffinose, D-turanose, and D-arabitol. Acid is not produced erythritol, D-arabinose, L-arabinose, L-xylose, adonitol, Methyl-βD-xylopyranoside, L-sorbose, L-rhamnose, dulcitol, inositol, N-acetylglucosamine, inuline, D-melezitose, amidon, glycogen, xylitol, gentiobiose, D-lyxose, D-tagatose, D-fucose, L-fucose, L-arabitol, potassium gluconate, potassium 2-ketogluconate, and potassium 5-ketogluconate. Cell-wall peptidoglycan contains *meso*-diaminopimelic acid. The predominant menaquinone is MK-7. The major cellular fatty acids are anteiso-C_15:0_ and C_16:0._ The polar lipid profile consists of phosphatidylglycerol, phosphatidylethanolamine, diphosphatidylglycerol, one unknown lipid, two unknown glycolipids and one unknown phospholipid. The DNA G+C content of strain MSt1^T^ is 51.5 mol%.

The type strain, MSt1^T^ (= DSM 100708^T^ = MCCC 1K01247^T^) was isolated from tropical peat swamp soil in North Selangor peat swamp, Malaysia. The 16S rRNA gene sequence of type strain MSt1^T^ has been deposited in DDBJ/EMBL/GenBank under the accession number KT216503.

## Author contributions

AYK performed the laboratory experiments, analyzed the data, and written up the manuscript. LSM supervised the entire study. LLH and CYL co-supervised the study. LSM, AYK, and OKS contributed to the experimental designs. LLH and AYK contributed to the polyphasic taxonomy. CYL and AYK contributed to the chemical analysis. CY, LSM, and AYK conceived the idea of bioprospecting in tropical peat swamp forest. All authors proofread and reviewed the manuscript.

## Funding

The authors express gratitude to External Industry Grant from Biotek Abadi (GBA-808138 & GBA-808813) awarded to Dr. LL for paying the article publication charges of this article.

### Conflict of interest statement

The authors declare that the research was conducted in the absence of any commercial or financial relationships that could be construed as a potential conflict of interest.
