# Supplementary material for: Newly Isolated Paenibacillus tyrfis sp. nov., from Malaysian Tropical Peat Swamp Soil with Broad Spectrum Antimicrobial Activity
Source: Front Microbiol. 2016 Mar 1;7:219. doi: 10.3389/fmicb.2016.00219 (PMC4771734; doi:10.3389/fmicb.2016.00219)
Supplement: Supplementary file 1 [file Image1.pdf]

Supplementary Material

Newly isolated Paenibacillus tyrfis sp. nov., from Malaysian tropical peat swamp soil with broad spectrum antimicrobial activity

Yoong-Kit Aw[1,3], Kuan-Shion Ong[1,3], Learn-Han Lee[2], Yuen-Lin Cheow[3], Catherine M. Yule[1,3] and Sui-Mae Lee[1,3*]

[1]Tropical Biology, Multidisciplinary Platform, School of Science, Monash University Malaysia, Jalan Lagoon Selatan, Bandar Sunway, 47500, Selangor, Malaysia

[2]Jeffrey Cheah School of Medicine and Health Sciences, Monash University Malaysia, Bandar Sunway, 47500, Selangor, Malaysia

[3]School of Science, Monash University Malaysia, Jalan Lagoon Selatan, Bandar Sunway, 47500, Selangor, Malaysia

Correspondence Email: lee.sui.mae@monash.edu

1 Supplementary Figures and Tables

Supplementary Figure S1. Scanning electron microscopy image of strain MSt1^T. Magnification of x10000 at 1kV. Cells approximately 3 – 5 µm in length and 0.6 – 1.0 µm in width

Supplementary figure S2: Polar lipid profile of strain MSt1^T on two-dimensional thin layer chromatography. DPG, diphosphatidylglycerol; PE, phosphatidylethanolamine; PG, phosphatidylglycerol; PL, unknown phospholipid; GL, unknown glycolipid; L, unknown lipid.

Supplementary Figure S3. Culture certificate for strain MSt1^T (DSM 100708) in DSMZ.

Supplementary Figure S4. Culture certificate for strain MSt1^T (MCCC 1K01247) in Marine Culture Collection of China.

1.1 Supplementary Figures

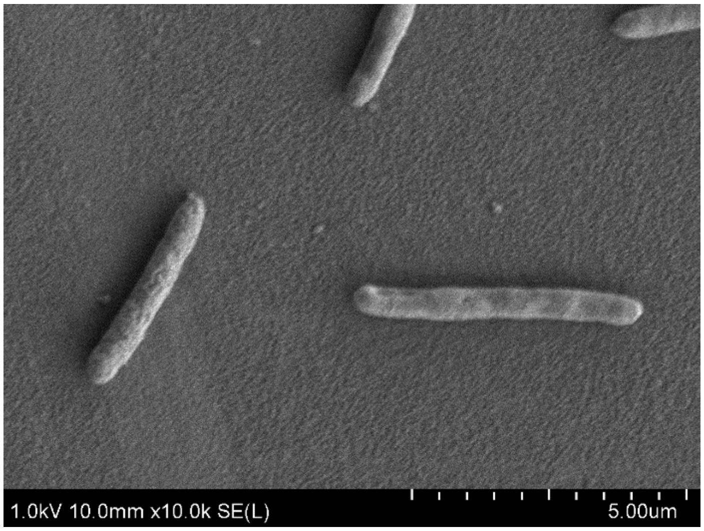

Supplementary figure S1: Scanning electron microscopy image of strain $MSt1^T$. Magnification of x10000 at 1kV. Cells approximately 3 – 5 µm in length and 0.6 – 1.0 µm in width

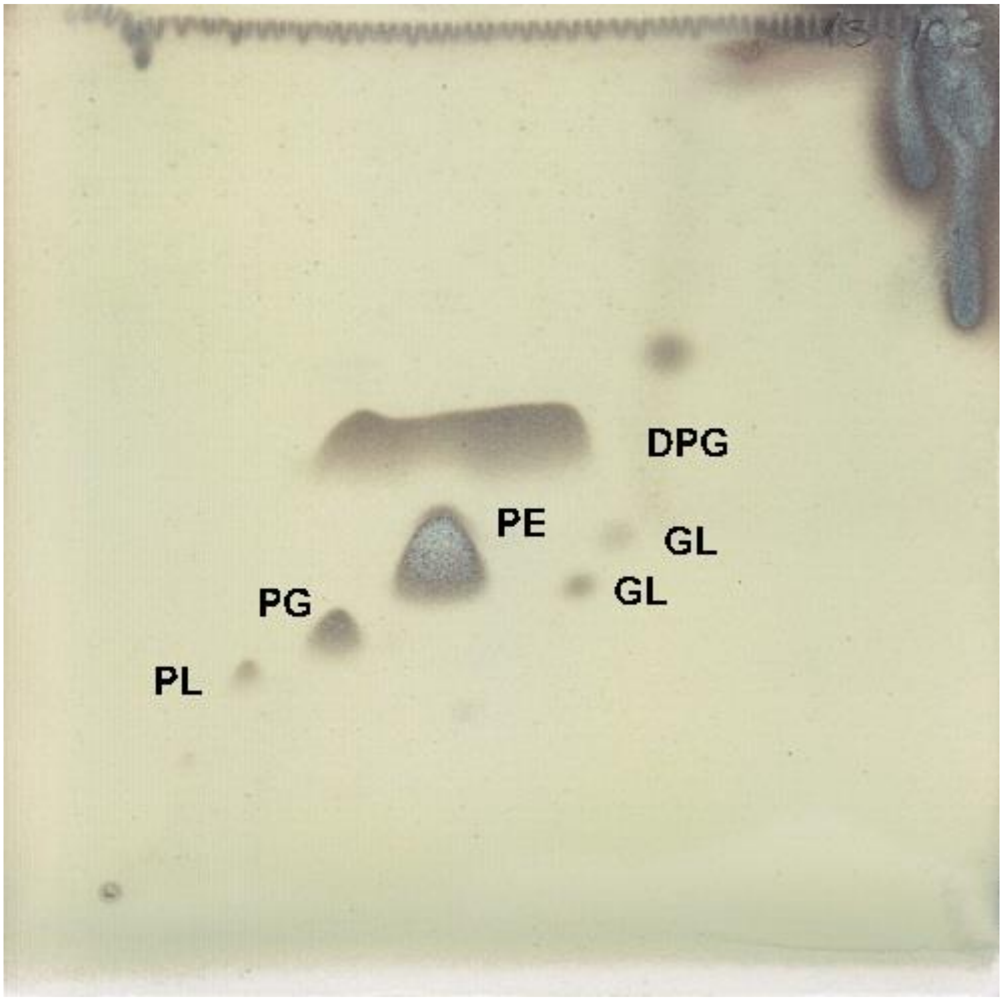

Supplementary figure S2: Polar lipid profile of strain MSt1^{T} on two-dimensional thin layer chromatography. DPG, diphosphatidylglycerol; PE, phosphatidylethanolamine; PG, phosphatidylglycerol; PL, unknown phospholipid; GL, unknown glycolipid; L, unknown lipid.

Leibniz-Institut
DSMZ-Deutsche Sammlung von
Mikroorganismen und Zellkulturen GmbH

Confirmation of the availability of a strain for the purpose of valid publication of a new name according to the Bacteriological Code

The following information is confidential and serves only to allow the International Journal of Systematic and Evolutionary Microbiology to confirm that a strain has been deposited and will be available from the DSMZ in accordance with the Rules of the Bacteriological Code (1990 revision) as revised by the ICSP at the plenary sessions in Sydney and Paris.

Strain Paenibacillus tyrfis MSt1 has been deposited in the DSMZ under the number

DSM 100708

This strain is available in the publicly accessible section of the DSMZ and restrictions have not been placed on access to information concerning the presence of this strain in the DSMZ. It will be included in published and online catalogues after publication of this number by the authors.

This strain has been checked for viability in the DSMZ and is stored using one of the standard methods used in the DSMZ. The depositor of this strain has also carried out a "depositor's check" and confirmed the identity of the strain held under this DSM number.

!! The DSMZ is not responsible for differences between the properties of the strain deposited in the DSMZ and properties given in the literature/databases !!

It is the sole responsibility of the depositor to ensure that type strains deposited in the DSMZ conform to the requirements of the appropriate Rules governing prokaryotes nomenclature and the deposition of type strains (Rules 18a, 27, & 30 of the ICNB/ICNP, including changes made at plenary sessions of the JC/ICSP).

Dr. R. Pukall, Curator Gram-positive Bacteria

Geschäftsführer/
Managing Director:
Prof. Dr. Jörg Overmann
Aufsichtsratsvorsitzender/Head of
Supervisory Board: RD Dr. David Schnieders

Braunschweigische Landessparkasse
(NORD/LB) Kto.-Nr./Account: 2 039 220
BLZ/Bank Code: 250 500 00
IBAN DE22 2505 0000 0002 0392 20
SWIFT (BIC) NOLADE 2 H

Handelsregister/
Commercial Register:
Amtsgericht Braunschweig
HRB 2570
Steuer-Nr. 13/200/24030

DAkkS
Deutsche
Akkreditierungsstelle
D-RM-18324-01-00

Supplementary figure S3: Culture certificate for strain $MSt1^T$ (DSM 100708) in DSMZ.

国家微生物资源平台

中国海洋微生物菌种保藏管理中心
Marine Culture Collection of China

CERTIFICATE OF DEPOSIT

IN MARINE CULTURE COLLECTION OF CHINA

Marine Culture Collection of China
Third Institute of Oceanography, State Oceanic Administration
Daxue Road 178, 361005 Xiamen, Fujian
P. R. China.
Phone/Fax: +86-592-2195177
Email: mccc5177@163.com
Web site: http://www.mccc.org.cn

MCCC 1K01247

***Paenibacillus tryfis* (strain Mst1) was received for deposit in Marine Culture Collection of China from**

Lee Learn-Han
Monash University Sunway Campus
Jalan Lagoon Selatan, 46150 Bandar Sunway, Selangor Darul Ehsan
Malaysia

on July 17, 2015

and was, after confirming the viability and purity, allocated the accession number MCCC 1K01247.

The strain is available to and *bona fide* individual, operating in a professional environment suitable for handling living material of the biohazard group involved.

Xiamen, Sept. 10, 2015

Dr. Zongze Shao
Public Collection Curator
Marine Culture Collection of China

Supplementary figure S4: Culture certificate for strain $MSt1^T$ (MCCC 1K01247) in Marine Culture Collection of China.
